# Elucidating the Relationship Between Diabetes Mellitus and Parkinson’s Disease Using ^18^F-FP-(+)-DTBZ, a Positron-Emission Tomography Probe for Vesicular Monoamine Transporter 2

**DOI:** 10.3389/fnins.2020.00682

**Published:** 2020-07-14

**Authors:** Yanyan Kong, Haicong Zhou, Hu Feng, Junyi Zhuang, Tieqiao Wen, Chencheng Zhang, Bomin Sun, Jiao Wang, Yihui Guan

**Affiliations:** ^1^PET Center, Huashan Hospital, Fudan University, Shanghai, China; ^2^Laboratory of Molecular Neural Biology, School of Life Sciences, Shanghai University, Shanghai, China; ^3^Department of Neurosurgery, Ruijin Hospital, Shanghai Jiao Tong University School of Medicine, Shanghai, China

**Keywords:** diabetes mellitus, β-cell mass, Parkinson’s disease, dopamine, VMAT2

## Abstract

Diabetes mellitus (DM) and Parkinson’s disease (PD) have been and will continue to be two common chronic diseases globally that are difficult to diagnose during the prodromal phase. Current molecular genetics, cell biological, and epidemiological evidences have shown the correlation between PD and DM. PD shares the same pathogenesis pathways and pathological factors with DM. In addition, β-cell reduction, which can cause hyperglycemia, is a striking feature of DM. Recent studies indicated that hyperglycemia is highly relevant to the pathologic changes in PD. However, further correlation between DM and PD remains to be investigated. Intriguingly, polycystic monoamine transporter 2 (VMAT2), which is co-expressed in dopaminergic neurons and β cells, is responsible for taking up dopamine into the presynaptic vesicles and can specifically bind to the β cells. Furthermore, we have summarized the specific molecular and diagnostic functions of VMAT2 for the two diseases reported in this review. Therefore, VMAT2 can be applied as a target probe for positron emission tomography (PET) imaging to detect β-cell and dopamine level changes, which can contribute to the diagnosis of DM and PD during the prodromal phase. Targeting VMAT2 with the molecular probe ^18^F-FP-(+)-DTBZ can be an entry point for the β cell mass (BCM) changes in DM at the molecular level, to clarify the potential relationship between DM and PD. VMAT2 has promising clinical significance in investigating the pathogenesis, early diagnosis, and treatment evaluation of the two diseases.

## Introduction

Parkinson’s disease (PD) is the most common progressive neurodegenerative disorder and is characterized by severe motor and non-motor symptoms including uncontrollable tremor, bradykinesia, rigidity, and sleep disturbances. More than 6 million individuals worldwide have PD (Armstrong and Okun, 2020). However, in the prodromal phase, diagnosis based on the clinical profiles is difficult. Diabetes mellitus (DM) is a metabolic disorder characterized by an absolute or relative deficiency of β cell mass (BCM), which manifests as persistent hyperglycemia (Jonietz, 2012; Cinti et al., 2016). DM can be largely classified into type I and type II. According to a 2019 epidemiological survey, the global prevalence of diabetes was an estimated 9.3% (463 million people) at publication, and it was predicted to rise to 10.2% (578 million) by 2030 and 10.9% (700 million) by 2045. The prevailing evidence points to diabetes accounting for a considerable global burden of chronic illness in aging societies (Saeedi et al., 2019; Sinclair et al., 2020).

Both PD and DM are highly prevalent. Previous research suggests that DM predisposes toward a Parkinson-like pathology and induces a more aggressive phenotype in patients already ill with PD (Pagano et al., 2018). In addition, studies have shown that these two diseases exhibit common pathogenic and pathological changes. Moreover, studies have demonstrated that hyperglycemia, which is caused by decreased BCM in DM patients, may lead to the occurrence of PD or aggravate PD symptoms.

The β-cell marker, vesicular monoamine transporter-2 (VMAT2), is closely linked to the occurrence of PD. Therefore, detecting progressive changes in BCM can both assist in the diagnosis of PD and clarify the pathogenesis of DM. Positron–emission tomography (PET) imaging using probes targeting VMAT2 has been applied to the diagnosis of PD and DM. Because of the low abundance of β cells, researchers use a notably sensitive tracer, an ^18^F-labeled dihydrotetrabenazine derivative (^18^F-FP-(+)-DTBZ), in their study (Wu et al., 2015). Researchers have used VMAT2 imaging with ^18^F-FP-(+)-DTBZ to identify DM as a risk factor for PD. In summary, this probe may, in the future, reveal the pathogenesis of DM and permit the early diagnosis of PD.

## Molecular Genetics, Cell Biology, and Epidemiology: Correlation Between PD and DM

Studies in molecular genetics, cell biology, and epidemiology have shown that the pathogeneses of PD and DM have common characteristics. Approximately 60% of patients with PD have impaired insulin signaling and impaired glucose tolerance (Santiago and Potashkin, 2014). Moreover, 62% of patients with PD and dementia have insulin resistance, and 30% of these patients also have impaired glucose tolerance (Bosco et al., 2012). PD is aggravated if the onset of comorbid DM is earlier than that of PD (Cereda et al., 2012). In the pathogenesis and progression of PD and DM, insulin and dopamine are regulated mutually, and hypoinsulinemia induced by streptozotocin can reduce the levels of dopamine transporter (DAT) and tyrosine hydroxylase (TH) transcription in the substantia nigra pars compacta (Lima et al., 2014). In addition, reduction of dopamine in the striatum can attenuate insulin signaling in the basal ganglia region. Pathways common to both diseases are mitochondrial dysfunction, endoplasmic reticulum stress, inflammatory response, vitamin D deficiency, and ubiquitin-protease/autophagy-lysosomal-system dysfunction (Table 1). Peroxisome proliferator-activated receptor gamma coactivator 1-alpha (PGC-1α) is a key regulator of the enzymes involved in the mitochondrial respiratory chain and of insulin resistance and plays an important role in the pathogenesis of both DM and PD. In addition, ATP-sensitive K^+^ channels, AMP-activated protein kinases, glucagon-like peptide-1, and dipeptidyl peptidase enzyme 4 show common pathological changes in DM and PD. Hyperglycemia can cause disturbances in the energy metabolism in the brain, damage neurons through various injury mechanisms, and lead to abnormal expression of proteins in the striatum and hippocampus. Consequently, recent studies have focused on the relationship between PD and hyperglycemia. One study found that thioredoxin-interacting protein (TXNIP), an endogenous inhibitor of reactive oxygen species elimination, regulates Parkin/PINK1-mediated mitophagy in dopaminergic neurons under high-glucose conditions (Su et al., 2020), which revealed the neuronal impact of mitochondrial dysfunction caused by hyperglycemia and hyperglycemia-induced oxidative stress. In addition, the preferential occurrence of nigrostriatal dopaminergic neurodegeneration in long-term hyperglycemia suggests that hyperglycemia causes premature aging of the central nervous system, fostering the development of age-related neurodegenerative diseases (Renaud et al., 2018). However, these studies did not sufficiently clarify the molecular link between hyperglycemia and PD. Further research on this topic will help elucidate the pathogenesis of PD, which is important for better clinical diagnosis, prevention, and treatment.

**TABLE 1:** Common pathogenesis pathways in Parkinson’s disease (PD) and diabetes mellitus (DM).

**Common pathways**	**Pathogenesis of PD**	**Pathogenesis of DM**
Mitochondrial dysfunction (Bonnard et al., 2008; Parker et al., 2008)	Increased ROS production—damage to lipids, protein, and DNA. Endoplasmic reticulum stress.	Increased ROS production, lipid accumulation. Endoplasmic reticulum stress. Insulin resistance.
Autophagy (Webb et al., 2003; Cuervo et al., 2004; Matos et al., 2018)	α-Synuclein aggregation. Lipid accumulation	Inclusion bodies in the liver and pancreas
Inflammatory response (Allan and Rothwell, 2001; Chen et al., 2008; Sun et al., 2011)	Increased production of cytokines IL-1β and TNF-α. Anti-inflammatory treatments are neuroprotective.	Chronic inflammation increases risk of diabetes. Anti-inflammatory treatments improve insulin resistance.
Metabolism (Moroo et al., 1994; Ogama et al., 2018; Ter Horst et al., 2018; Wang et al., 2020)	60–80% of PD patients exhibit impaired glucose tolerance. Dopamine release is glucose sensitive Loss of insulin-receptor immunoreactivity in the substantia nigra.	Insulin resistance is associated with cognitive decline. Peripheral insulin resistance leads to ischemic cerebrovascular disease. Hyperglycemia is associated with neurodegeneration.
Vitamin D deficiency (Derex and Trouillas, 1997; Boucher et al., 2004; Evatt et al., 2008)	Reduced vitamin D levels increase the risk of PD. Vitamin D improves motor function in human PD	Reduced vitamin D levels increase the risk of diabetes. Vitamin D improves insulin resistance in diabetes.

Both type 2 diabetes (T2DM) and PD are involved in the accumulation of misfolded proteins in amyloid aggregates. In diabetic patients, the accumulation of islet amyloid polypeptide (IAPP) in pancreatic cells can cause cell dysfunction. In patients with PD, α-synuclein eventually aggregates into the Lewy bodies (Wang and Raleigh, 2014; Bridi and Hirth, 2018). Researchers have found that IAPP and α-synuclein cross-interact in the two diseases, and IAPP in patients with T2DM can promote α-synuclein aggregation, which leads to the occurrence of PD (Horvath and Wittung-Stafshede, 2016). Another study showed that insulin-degrading enzymes (IDE) can bind to synuclein oligomers to prevent further aggregation. Moreover, insulin resistance can inhibit IDE and promote the formation of α-synuclein fibrils, which may enhance PD progression (Sharma et al., 2015).

1-Methyl-4-phenyl-1,2,3,6-tetrahydropyridine (MPTP) can lead to neuronal death by inhibiting the mitochondrial respiratory chain enzyme, which is a PD animal model inducer (Jing et al., 2017). In addition, studies have shown that mitochondrial dysfunction occurs in patients with DM and PD, and insulin resistance in diabetic mice can lead to mitochondrial destruction and dopaminergic neuron degeneration (Khang et al., 2015).

According to many case–control studies, longitudinal studies, and meta-analyses, DM is a risk factor for PD (some of these results are shown in Table 2). DM can accelerate the development and progression of PD, especially in young women aged between 8 and 12 years (Sun et al., 2012; Aviles-Olmos et al., 2013a; Ahn et al., 2015; Santiago et al., 2017; Pagano et al., 2018). Several studies in the late 1990s showed that, in the diabetic mouse, the levels of DAT in the V9 and V10 regions (Del Pino et al., 2017) and in the medial forebrain bundle of the midbrain (Petrisic et al., 1997) were decreased significantly. A positive correlation was established between insulin reduction in DM and decreases in the levels of DAT, VMAT (mainly VMAT2), and TH. Insulin regulates DAT and VMAT2 levels through the PI3K/AKT pathway, and insulin deficiency leads to DAT and VMAT2 dysfunction. Dopamine D2 receptors can also regulate DAT and VMAT2 expression through the ERK signaling pathway (Carvelli et al., 2002; Owens et al., 2012; Samandari et al., 2013; Bini et al., 2019).

**TABLE 2:** Recent studies on the correlation between diabetes mellitus (DM) and Parkinson’s disease (PD).

**Study**	**Study design**	**Sample size**	**Main results**
Hu et al., 2007	Cohort	PD: 633 Controls: 51,552	T2DM is associated with an increased risk of PD.
Moran and Graeber, 2008	Meta-analysis	N/A	Shared biological pathways between PD, T2DM, cancer, and inflammation.
D’Amelio et al., 2009	Case–control	PD: 318 Controls: 318	Inverse association between PD and DM preceding PD onset.
Palacios et al., 2011	Case–control	PD: 1931 Controls: 9651	T2DM is associated with an increased risk of PD, especially younger-onset PD.
Xu et al., 2011	Cohort	DM: 21,611 Controls: 267,051	T2DM is associated with an increased risk of PD.
Bosco et al., 2012	Case–control	PD+dementia: 53 PD: 57	Insulin resistance is associated with an increased risk of dementia in PD.
Sun et al., 2012	Case–control	DM: 603,416 Controls: 472,118	DM is associated with an increased risk of PD onset.
Wahlqvist et al., 2012	Case–control	DM: 64,166 Controls: 698,587	T2DM is associated with an increased risk of PD. Metformin-sulfonylurea therapy reduces the risk of PD.
Yue et al., 2016	Meta-analysis	Based studies: 7 Sample total: 1,761,632	Diabetes is associated with an ∼38% increase in the risk of PD.
De Pablo-Fernandez et al., 2017	Cohort	PD: 79 Controls: 4919	Diabetes duration might be an important factor in the association of PD and diabetes.
De Pablo-Fernandez et al., 2018	Cohort	DM: 2,017,115 Controls: 6,173,208	Significantly elevated rates of PD following T2DM.
Pagano et al., 2018	Case–control	PD+DM: 25 PD without DM: 25 DM: 14 Controls: 14	DM may predispose toward a Parkinson-like pathology and, when present in patients with PD, can induce a more aggressive phenotype.

*T2DM, type 2 diabetes mellitus.*

There are also other molecular targets that tightly link PD and DM. For example, glucose-dependent insulin-promoting polypeptide (GIP) is not only an endogenous hormone of the incretin family but also a neurotrophic factor. It activates cell proliferation and protects the neurons by promoting cell repair and preventing apoptosis, which enhances the survival ability of β-cells in the pancreas and neurons in the brain (Maino et al., 2014; Altmann et al., 2016). Besides, the GIP receptor is a G protein-coupled receptor that belongs to the glucagon family and increases insulin secretion during the onset of hyperglycemia (Park et al., 2013). In addition, studies have revealed that treatment with GIP analogs can increase the level of reduced TH in PD patients (Li et al., 2016).

Another incretin hormone, glucagon-like peptide-1 (GLP-1), which is functionally similar to GIP, also plays a role in DM and PD pathogenesis. The GLP-1 receptor agonists have been approved for the treatment of T2DM and have a good therapeutic effect on the clinical symptoms of PD including motor and cognitive dysfunctions. Their neuroprotective effect is shown in the protection of dopaminergic neurons in the substantia nigra, and they reduce the accumulation of synuclein proteins (Aviles-Olmos et al., 2013b; Ji et al., 2016). In addition, GLP-1 treatment reduces oxidative stress and lipid peroxidation and increases the level of brain-derived neurotrophic factor (BDNF), alleviating the symptoms of neuroinflammation in the brain (Li et al., 2017). Studies on the effects of GLP1 analogs on MPTP rodents have revealed that by inhibiting inflammation and excitatory cytokines and stimulating antioxidant enzymes, striatum dopamine levels can be improved and neuronal damage can be reduced (Elbassuoni and Ahmed, 2019).

After treatment with GLP1/GIP receptor agonists, TH in the brain increases and microglial activation decreases, promoting the production of dopamine and providing neuroprotection for dopaminergic neurons. These two peptides can be used as evidence for the connection between PD and DM.

Although these studies proved that DM is a risk factor for PD, the mechanistic basis of the relationship has not been fully elucidated. In addition, because a deficiency in BCM is the absolute or relative characteristic of DM, early, effective evaluation of BCM levels in patients with DM will be of great significance for the prevention, early diagnosis, and treatment of PD. VMAT2 is expressed in brain monoaminergic neurons, especially in the striatum, which is a regulator of monoaminergic neuronal function. Besides, VMAT2 is also a potential target for the assessment of BCM, and decreases in BCM level in the human pancreases correlate with decreases in insulin level in the blood (Anlauf et al., 2003; Fu et al., 2019). Jiang et al. (2020) found a decreased VMAT2 expression in the striatum due to the β-cell impairment in a type 1 DM (T1DM) rat model. Furthermore, VMAT2 expression in the striatum was also increased with the recovery of BCM and glucose levels in DM rats after streptozotocin injection treatment (Jiang et al., 2020). VMAT2 is, therefore, a key factor in connecting PD and DM.

Considering the correlation between DM and PD, researchers have employed a molecular imaging probe for VMAT2, the PET radioligand [18F]fluoropropyl-(+)-dihydrotetrabenazine (18F-FP-(+)-DTBZ or 18F-AV-133), as a molecular marker that can be used to objectively analyze dopamine and β cells.

## VMAT2 Function and Anatomical Distribution

VMAT2, a glycoprotein bound to secretory vesicle membranes and a constitutive protein in humans, is a subtype of the vesicle monoamine transporter (Stahl, 2018). A large number of studies have shown that VMAT2 is pivotal in the pathogenesis of PD (Ma et al., 2019; Shi et al., 2019), which is well known as a disease of dopamine deficiency. In endocrine islets, a large number of genes expressed by β cells have homologs in neural cells, one of which is VMAT2 (Harris et al., 2008). In 2003, Anlauf et al. (2003) determined the distribution of VMAT2 in human and monkey pancreas by immunohistochemistry and *in situ* hybridization. This was the first confirmation of VMAT2 localized in islet β cells. The monoamine neurotransmitters transported by VMAT2 are important paracrine and/or autocrine regulators of islet β cells and include dopamine, which inhibits insulin release via dopamine D2 receptors on β cells (Pecic et al., 2019). Recent studies have found that VMAT2 also mediates the release of the inhibitory neurotransmitter γ-aminobutyric acid (Tritsch et al., 2012), which plays a protective role by inhibiting β-cell apoptosis (Purwana et al., 2014). Exocytosis or endocytosis of the vesicles is important for the regulation of cellular signal transduction. Although glucose is an important factor in stimulating insulin secretion, VMAT2 also regulates glucose and insulin homeostasis (Raffo et al., 2008; Rodriguez-Diaz et al., 2011). Neuroendocrine cells accumulate biogenic amines and peptides, and the VMAT2 that they express serves to take up monoamines from the cytoplasm, which are then stored in the secretory vesicles for use in signaling (Bernstein et al., 2012). Accumulating evidence shows that VMAT2 is a contributor in PD progression (Ma et al., 2019; Shi et al., 2019). The accumulation of toxic α-synuclein in the prodromal phase of PD can impair VMAT2 activity, which eventually increases the cytosolic levels of dopamine (Bridi and Hirth, 2018). The metabolites of dopamine itself are cytotoxic; thus, an abnormal level of dopamine can exacerbate PD symptoms (Lotharius and Brundin, 2002). Moreover, disturbances of VMAT2 expression decrease neurogenesis in the olfactory nerve, causing hyposmia, which is another symptom of PD (Ma et al., 2019). It is known that the main histopathological change observed in PD is the selective degeneration of dopaminergic neurons in the substantia nigra pars compacta and of dopaminergic nerve endings in the striatum. A previous study identified a correlation between decreased striatal VMAT2 and multiple non-motor symptoms in patients with PD (Shi et al., 2019).

Furthermore, physiological effects of knocking out the VMAT2 mouse model were studied. Homozygous (VMAT2-/-) mice die within a few days after birth due to poor eating. The number of monoaminergic cells in the brain of knockout mice is lower than that of wild-type littermates (Fon et al., 1997). In addition, heterozygosity (VMAT2 +/−) shows low sensitization to dopamine agonist synaptic apomorphine, psychostimulant cocaine, and other neurotoxins (Wang et al., 1997). In VMAT2 knockout mice, disruption of vesicle trafficking leads to enhanced methamphetamine (METH)-induced dopaminergic neurotoxicity. METH produces greater dopamine consumption and metabolite content in the VMAT2 +/− striatum. DAT expression in the striatum of VAT2 +/− mice was further reduced, and the dopamine transporter content was lower than that of the wild type after treatment with METH (Fumagalli et al., 1999). Moreover, neurotoxin MPTP to heterozygotes’ dopamine cells is more than two times lower than that in wild-type mice (Takahashi et al., 1997). These results suggest that monoamine function, post-synaptic sensitization, and neurotoxin mechanism of action are linked to VMAT2 expression. In summary, VMAT2 may play an important role in the pathogenesis of PD.

**FIGURE 1:**
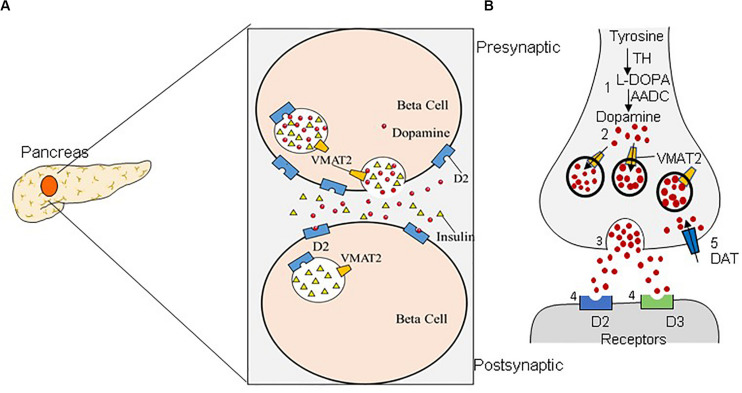
Dual distribution of the vesicular monoamine transporter-2 (VMAT2). VMAT2 is expressed in both β cells **(A)** and dopaminergic neurons **(B)**. **(A)** In pancreatic β cells, VMAT2 can accumulate cytoplasmic insulin in secretory vesicles. **(B)** VMAT2 is also responsible for accumulating cytoplasmic dopamine in synaptic vesicles in the classical dopaminergic neuron in the central nervous system.

T1DM is caused by the reductions in BCM due to an autoimmune reaction, resulting in an absolute deficiency of endogenous insulin secretion (Wilcox et al., 2016; Yu et al., 2019). T2DM stems from metabolic disorders and insulin resistance, and the toxic downstream effects of these metabolic disorders can further exacerbate BCM reduction by up to 60% (Ashcroft and Rorsman, 2012; Johnson and Olefsky, 2013; Derakhshan et al., 2015). Significant losses of BCM precede rises in blood glucose; therefore, an in-depth study of β-cell numbers can provide new clues to the pathology, early diagnosis, and treatment of DM. A non-invasive method for determining the residual BCM of patients with DM would be extremely useful. Great efforts have been devoted to improving the diagnostic accuracy of DM/BCM determination using highly β-cell-specific molecular imaging probes. Considering the necessity of tracking dynamic changes in BCM over time, PET imaging seems to be a feasible method for accessing BCM (Wei et al., 2019). Moreover, VMAT2 is one of the β-cell biomarkers, and the binding of VMAT2 with specific radioactive probe substances is well correlated with the amount of insulin secretion observed after a glucose challenge (Naganawa et al., 2018). Compared with the approach of directly determining insulin levels, determining VMAT2 as a proxy for BCM has the advantages of not being affected by insulin secretion, metabolism, inflammation, etc. (Freeby et al., 2012). In addition, multiple molecular probes are available for imaging β cells, including the voltage-dependent Ca^2+^ channel (VDCC), the glucose transporter (GLUT), the radiotracer ^18^F-fluorodeoxyglucose (^18^F-FDG), VMAT2, dihydrotetrabenazine (DTBZ), 5-hydroxytryptophan (5-HTP), and GPL-1 receptor (GLP-1R) (Wei et al., 2019). VMAT2 has high specific binding and a high density in β cells, which makes it a promising target for BCM imaging (Yang et al., 2017). Dopaminergic neuron damage in PD primarily begins with the nigrostriatal neurons, subsequently affecting the nigrostriatal pathway and finally the mesolimbic system. The striatum has the highest VMAT2 level (Hsiao et al., 2014). VMAT2 is located in the presynaptic neurons, which are responsible for storing and packing the neurotransmitter to regulate the cytoplasmic dopamine level (Figure 1; Hefti et al., 2010). Moreover, previous studies have delineated a strong correlation between the density of striatal VMAT2 and the non-motor symptoms of PD (Shi et al., 2019). Therefore, VMAT2 is promising as an imaging target for the diagnosis PD.

## Imaging the Monoaminergic System: VMAT2

### Role in PD Research

**FIGURE 2:**
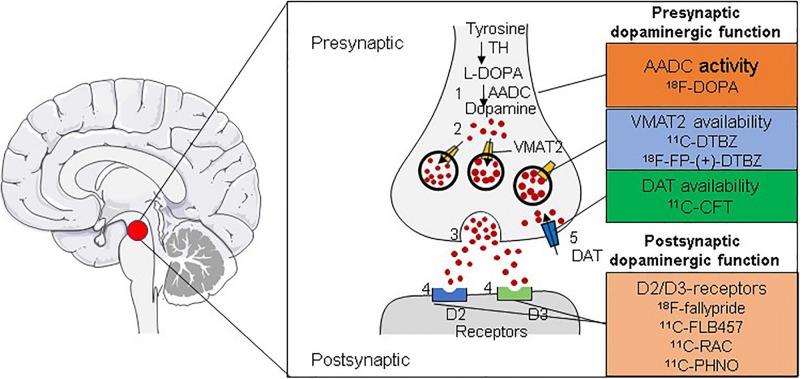
Radiotracers for imaging the dopaminergic system. Shown are the radiotracers used for the *in vivo* imaging of the dopaminergic system for the diagnosis of PD. Tracers can be used for imaging either presynaptic (AADC, VMAT2, or DAT) or post-synaptic (D2/D3 receptors) dopaminergic function through positron–emission tomography (PET).

At present, the positron–emission molecular probes used in the diagnosis of PD mainly employ dopaminergic targets and are divided into presynaptic and post-synaptic imaging agents according to their binding targets. Targets on the presynaptic membrane include the following: (1) The dopamine transporter (DAT) of dopaminergic neurons, which transports DA from the synaptic gap back to the presynaptic compartment. Its signal is closely related to the number of dopaminergic neurons. Therefore, DAT imaging can assess the function of dopaminergic nerve endings. ^11^C-methyl-N-2b-carbomethoxy-3b-(4-fluorophenyl)tropane (^11^C-CFT) is a DAT radiotracer widely used in clinical practice. However, DAT is commonly downregulated in the early stages of disease, resulting in an overestimation of the amount of degeneration (Lee et al., 2000). (2) L-Aromatic amino acid decarboxylase (AADC) is one of the most important enzymes in dopamine synthesis, being responsible for converting levodopa into dopamine. The activity of AADC is measured by the tracer L-3,4-dihydroxy-6-^18^F-fluorophenylalanine (^18^F-DOPA) (Stormezand et al., 2020). (3) VMAT2 is responsible for taking up cytoplasmic dopamine into presynaptic vesicles. The main tracers used are ^11^C-dihydrotetrabenazine (^11^C-DTBZ) and ^18^F-FP-(+)-DTBZ (Bohnen et al., 2006). Furthermore, post-synaptic dopamine receptors are mainly in the D1 family (D1 and D5) and D2 family (D2, D3, and D4). The main tracers used are ^11^C-FLB 457, ^18^F-fallypride, and ^11^C-raclopride (^11^C-RAC), which bind to D2 (Laruelle, 2000; Ray et al., 2012), and ^11^C-PHNO, which binds to D3 (Figure 2; Payer et al., 2016).

The diagnostic imaging agent most used to assess DAT expression in cases of PD is ^11^C-CFT, but its use is limited by the short half-life of ^11^C (20 min). Recent studies have shown that, in the primate PD model, the changes in DAT and VMAT2 binding sites in the striatum of surviving substantia nigra neurons are similar, suggesting that targeting VMAT2 with the ^18^F-labeled dihydrotetrabenazine derivative (^18^F-FP-(+)-DTBZ) for imaging can provide results similar to those of the DAT imaging presently used to diagnose PD (Lin et al., 2014; Wood, 2014; Cho et al., 2019), but with the further advantage that ^18^F-FP-(+)-DTBZ imaging can also be used to assess the severity of PD (Hsiao et al., 2014). Another advantage is that it is less affected by compensation or pharmacological regulation (Wilson and Kish, 1996).

### ^18^F-FP-(+)-DTBZ in the Diagnosis of DM

^11^C-Dihydrotetrabenazine (^11^C-DTBZ) is a specific VMAT2 radioligand currently used in PD research and in clinical imaging of the brain for the diagnosis of PD (Lin et al., 2013). In rodents, a model of type-1 DM showed a good correlation between ^11^C-DTBZ uptake in the pancreas and blood glucose homeostasis (Naganawa et al., 2016).

A limitation of PET technology in VMAT2 imaging is its low spatial resolution. Moreover, β cells account for a small proportion (1–2%) of the pancreas and are relatively dispersed. To overcome these challenges, it is necessary to further improve the signal-to-noise ratio of the images provided by the radiotracer. Therefore, the ^18^F-labeled dihydrotetrabenazine derivative ^18^F-FP-(+)-DTBZ (^18^F-AV-133), which improves upon certain properties of ^11^C-DTBZ, is preferred. Compared with ^11^C-DTBZ, ^18^F-FP-(+)-DTBZ has better affinity for VMAT2 and lower fat solubility, which translates into less non-specific binding (Wu et al., 2015). In addition, ^18^F has a longer half-life than ^11^C, making it suitable for a wider range of applications.

In the most recent primate experiments, the renal cortex, which contains no VMAT2, has been used as a control for non-specific binding in estimations of the specific binding ability of ^18^F-FP-(+)-DTBZ to VMAT2. Recent experiments on sputum have confirmed that the probe’s specific binding capacity to such samples can reach 85% of that in the striatum, and pancreatic BCM has been successfully reevaluated by PET scanning in humans (Naganawa et al., 2016). ^18^F-FP-(+)-DTBZ can be both precise and accurate. A quantitative display of PET results, therefore, can be used as a non-invasive means to effectively quantify the BCM and thus the secretion of insulin, which has broad application in the diagnosis, treatment, and monitoring of DM (Singhal et al., 2011). The ^18^F-FP-(+)-DTBZ-standardized uptake value (SUV), the total volume of distribution, and the binding potential in the pancreas were shown to be reduced by 38, 20, and 40%, respectively, in patients with T1DM compared with those in healthy controls (Normandin et al., 2012). Moreover, the new procedure reduced the patient’s exposure to radioactivity. In addition, the extent of tracer uptake correlated with the rate of insulin secretion. The PET Center of Huashan Hospital in China conducted a study in baboons on the use of ^18^F-FP-(+)DTBZ for imaging the pancreas and reached similar conclusions (Naganawa et al., 2016).

### VMAT2 Imaging Reveals a Correlation Between PD and DM

Using ^18^F-FP-(+)DTBZ imaging of rat models of T1DM and T2DM, previous studies at the Huashan Hospital PET Center demonstrated that the uptake of ^18^F-FP-(+)DTBZ in the striatum and the fasting blood glucose of the two groups were significantly negatively correlated. This indicates that BCM is closely related to VMAT2 expression in the brain. At the same time, T2DM caused a decrease in the expression of VMAT2 in the dopaminergic pathway in the brain. In addition, it was found that in the dopamine neurological abnormalities caused by T2DM, the abnormal expression of VMAT2 appeared earlier than that of DAT, and to a greater degree. These results suggest that DM and PD have a common pathogenesis.

**FIGURE 3:**
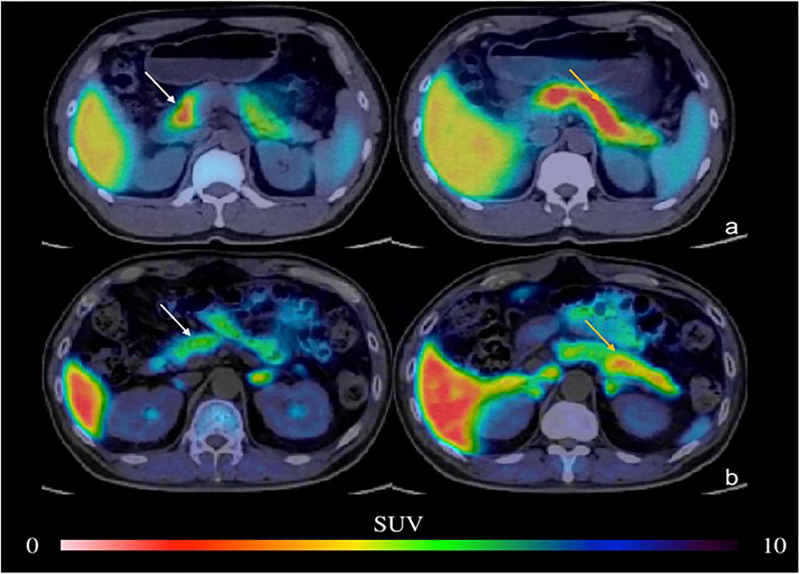
PET imaging of the pancreas in type 2 DM (Donglang et al., 2018) (E-produced/adapted from Chin J Endocrinol Metab, used with permission) Representative PET imaging of the pancreatic head, body, and tail in the baboon in type 2 DM and in healthy controls. **(a)** Normal pancreas; **(b)** type 2 DM; white arrow, pancreatic head; yellow arrow, pancreatic body and tail; SUV, standardized uptake value.

**FIGURE 4:**
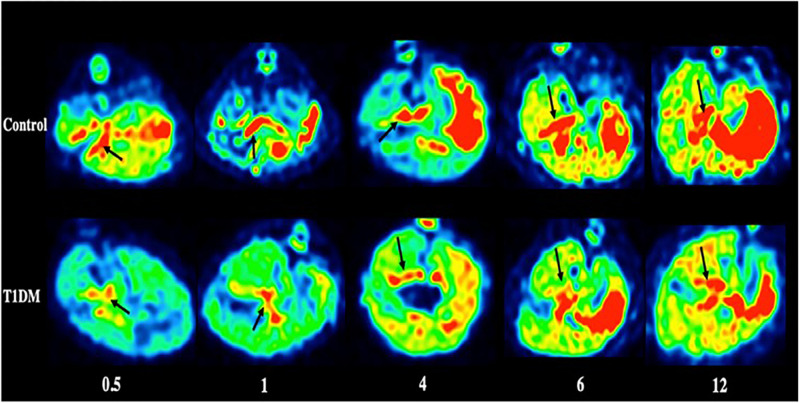
PET imaging of the pancreas in type 1 DM (Jianfei et al., 2019) (E-produced/adapted from Chin J Endocrinol Metab, used with permission) Shown are representative ^18^F-PF-(+)-DTBZ PET images of the pancreas (black arrows) of rats with type 1 diabetes mellitus (T1DM) and control rats, 0.5, 1, 4, 6, and 12 months after model induction.

**FIGURE 5:**
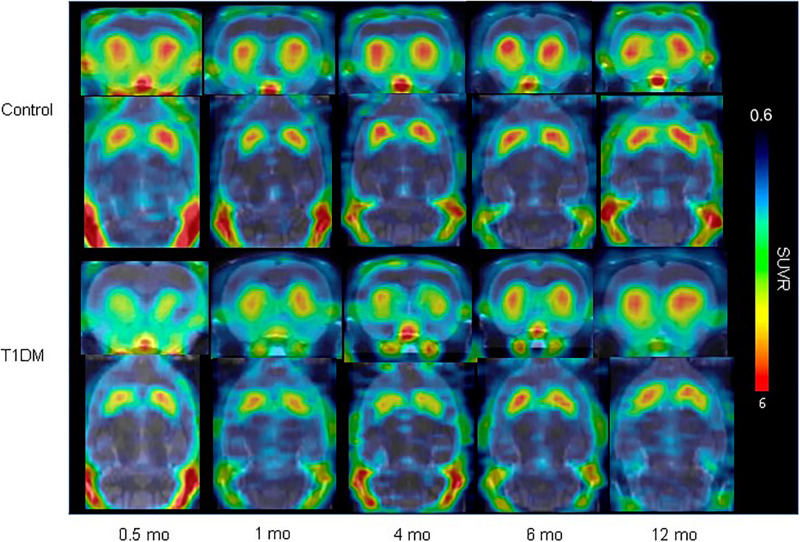
VMAT2 expression in the striatum in type 1 DM (Jiang et al., 2020) (E-produced/adapted from Nuclear Medicine and Biology, used with permission) Shown is the decreased VMAT2 expression in the striatum in a rat model of T1DM, and in control rats, 0.5, 1, 4, 6, and 12 months after model induction, as imaged by ^18^F-FP-(+)-DTBZ-PET/CT; SUVR, standardized uptake ratio.

Furthermore, imaging of the pancreas in patients with T1DM, patients with T2DM, and normal controls revealed that the uptake of ^18^F-FP-(+)-DTBZ was significantly lower in T1DM and T2DM than in controls (Figures 3, 4; Donglang et al., 2018; Jianfei et al., 2019). Quantification of ^18^F-FP-(+)-DTBZ can be used to evaluate BCM in the pancreas of diabetic patients. ^18^F-FP-(+)-DTBZ imaging in the caudate nucleus and putamen of patients with comorbid PD and T2DM showed that the standardized uptake value ratio (SUVR) of the caudate nucleus in the comorbid group was significantly lower than that of PD patients and normal controls. The SUVR was also lower in the putamen in the comorbid group than in PD patients. This result indicates that T2DM exacerbates the decline in VMAT2 expression in the caudate and putamen of the brain (Figure 5; Jiang et al., 2020). Previous clinical studies have also found that DM aggravates the symptoms of PD (Yue et al., 2016).

As a protein that transports neurotransmitters, the distribution and expression of VMAT2 in human pancreas and its presence in sputum cells have clinical applications. The homology of the human, mouse, and rat VMAT2 amino acid sequences can be above 90% (German et al., 2015; Wu et al., 2016). Thus, studies based on diverse animal models suggest a correlation between BCM reduction and PD in humans (Jiang et al., 2020). In summary, diabetes is a demonstrated risk factor for PD, and a correlation between DM and PD has been established by the above experiments.

## Future Perspectives

The physiological function of VMAT2 in islet β cells needs further study to determine the molecular mechanisms affecting BCM and insulin secretion and how VMAT2 participates in regulating the secretion of neurotransmitters. This information will provide new molecular therapeutic targets and an important theoretical basis for the early prediction, diagnosis, and treatment of DM. Future research should use PET scans to compare VMAT2 distributions among patients with T2DM, patients with obesity but without DM, and participants without DM. Undoubtedly, the advantages of using VMAT2 as a BCM marker will lead to useful information.

Positron emission tomography has shown prominent features compared with other molecular imaging approaches. PET can trace suspicious malignant lesions and provide functional imaging of those areas, which is useful for early diagnosis of diseases (Kang et al., 2004; Machtens et al., 2004). Although magnetic resonance imaging (MRI) has a higher resolution, the specificity of its related contrast agents for β cells is not as good as PET imaging. Moreover, the radiation exposure of PET imaging is much lower than that of CT scan. In addition, the evaluation of the BCM level has a significant effect through PET imaging (Wei et al., 2019; Yang et al., 2019). However, the high cost of PET has limited its promotion.

Molecular imaging allows qualitative and quantitative studies of biological processes *in vivo* at the cellular and molecular levels. The establishment of a functional imaging platform based on PET technology to observe physiological and pathological changes in the BCM can objectively, intuitively, and quantitatively reveal the key factors affecting insulin secretion and insulin resistance. Such a platform will advance our understanding of the pathogenesis of DM and the evaluation of the efficacy of experimental treatments, resulting in great clinical and social benefits.

## Conclusion

Targeting VMAT2 with the molecular probe ^18^F-FP-(+)-DTBZ provides an entry point for revealing the relationship between DM and PD. Interest in the use of imaging methods in research on BCM determination and DM has steadily increased because of their practicality, non-invasiveness, and safety. At present, radionuclide labeling remains the most sensitive imaging method for human β cells. This approach serves to clarify the role of BCM in the pathogenesis and progression of DM and has practical, clinical, and social value for the early diagnosis and treatment of DM.

## Author Contributions

YK conceived and designed the idea for the review. YK, HZ, HF, and JZ searched and reviewed the PD and DM literature and drafted the manuscript. YK, JW, and CZ further revised the manuscript. JW, YG, and BS reviewed and edited the manuscript. All authors read and approved the final manuscript.

## Conflict of Interest

The authors declare that the research was conducted in the absence of any commercial or financial relationships that could be construed as a potential conflict of interest.

## Abbreviations

^11^C-CFT: ^11^C-methyl-N-2b-carbomethoxy-3b-(4-fluorophenyl) tropane
^11^C-DTBZ: ^11^C-dihydrotetrabenazine
^11^C-DTBZ: ^11^C-dihydrotetrabenazine
^11^C-RAC: ^11^C-raclopride
^18^F-DOPA: L-3,4-dihydroxy-6-^18^F-fluorophenylalanine
^18^F-FP-(+)-DTBZ: ^18^F-fluoropropyl-(+)-dihydrotetrabenazine
AADC: L-aromatic amino acid decarboxylase
BCM: β cell mass
DA: dopamine
DAT: dopamine transporter
DM: diabetes mellitus
DTBZ: dihydrotetrabenazine
PD: Parkinson’s disease
PET: positron-emission tomography
SUVR: standardized uptake value ratio
T1DM: type 1 DM
T2DM: type 2 DM
TH: tyrosine hydroxylase
VMAT2: vesicular monoamine transporter-2.
