# Supplementary material for: Elucidating the Relationship Between Diabetes Mellitus and Parkinson’s Disease Using 18F-FP-(+)-DTBZ, a Positron-Emission Tomography Probe for Vesicular Monoamine Transporter 2
Source: Front Neurosci. 2020 Jul 14;14:682. doi: 10.3389/fnins.2020.00682 (PMC7372188; doi:10.3389/fnins.2020.00682)
Supplement: Supplementary file 1 [file Table_1.docx]

**Table 1.** Common pathogenesis pathways in Parkinson’s disease (PD) and diabetes mellitus (DM)

| **Common pathways** | **Pathogenesis of PD** | **Pathogenesis of DM** |
| --- | --- | --- |
| Mitochondrial dysfunction [[1](#_ENREF_1), [2](#_ENREF_2)] | Increased ROS production – damage to lipids, protein, and DNA. Endoplasmic reticulum stress. | Increased ROS production, lipid accumulation.  Endoplasmic reticulum stress.  Insulin resistance. |
| Autophagy [[3-5](#_ENREF_3)] | α-Synuclein aggregation.  Lipid accumulation | Inclusion bodies in the liver and pancreas |
| Inflammatory response [[6-8](#_ENREF_6)] | Increased production of cytokines IL-1β and TNF-α.  Anti-inflammatory treatments are neuroprotective. | Chronic inflammation increases risk of diabetes.  Anti-inflammatory treatments improve insulin resistance. |
| Metabolism[[9-12](#_ENREF_9)] | 60–80% of PD patients exhibit impaired glucose tolerance.  Dopamine release is glucose sensitive  Loss of insulin-receptor immunoreactivity in the substantia nigra. | Insulin resistance is associated with cognitive decline.  Peripheral insulin resistance leads to ischemic cerebrovascular disease.  Hyperglycemia is associated with neurodegeneration. |
| Vitamin D deficiency[[13-15](#_ENREF_13)] | Reduced vitamin D levels increase the risk of PD.  Vitamin D improves motor function in human PD | Reduced vitamin D levels increase the risk of diabetes.  Vitamin D improves insulin resistance in diabetes. |

1. Bonnard, C., et al., *Mitochondrial dysfunction results from oxidative stress in the skeletal muscle of diet-induced insulin-resistant mice.* J Clin Invest, 2008. **118**(2): p. 789-800.

2. Parker, W.D., Jr., J.K. Parks, and R.H. Swerdlow, *Complex I deficiency in Parkinson's disease frontal cortex.* Brain Res, 2008. **1189**: p. 215-8.

3. Webb, J.L., et al., *Alpha-Synuclein is degraded by both autophagy and the proteasome.* J Biol Chem, 2003. **278**(27): p. 25009-13.

4. Matos, M., et al., *Pancreatitis Is an Important Feature of Broilers Suffering from Inclusion Body Hepatitis Leading to Dysmetabolic Conditions with Consequences for Zootechnical Performance.* Avian Dis, 2018. **62**(1): p. 57-64.

5. Cuervo, A.M., et al., *Impaired degradation of mutant alpha-synuclein by chaperone-mediated autophagy.* Science, 2004. **305**(5688): p. 1292-5.

6. Chen, H., et al., *Peripheral inflammatory biomarkers and risk of Parkinson's disease.* Am J Epidemiol, 2008. **167**(1): p. 90-5.

7. Sun, X., et al., *Effect of aspirin on the expression of hepatocyte NF-kappaB and serum TNF-alpha in streptozotocin-induced type 2 diabetic rats.* J Korean Med Sci, 2011. **26**(6): p. 765-70.

8. Allan, S.M. and N.J. Rothwell, *Cytokines and acute neurodegeneration.* Nature Reviews Neuroscience, 2001. **2**(10): p. 734-744.

9. Wang, C., et al., *High Plasma Resistin Levels Portend the Insulin Resistance-Associated Susceptibility to Early Cognitive Decline in Patients with Type 2 Diabetes Mellitus.* J Alzheimers Dis, 2020.

10. Ter Horst, K.W., et al., *Striatal dopamine regulates systemic glucose metabolism in humans and mice.* Sci Transl Med, 2018. **10**(442).

11. Ogama, N., et al., *Postprandial Hyperglycemia Is Associated With White Matter Hyperintensity and Brain Atrophy in Older Patients With Type 2 Diabetes Mellitus.* Front Aging Neurosci, 2018. **10**: p. 273.

12. Moroo, I., et al., *Loss of insulin receptor immunoreactivity from the substantia nigra pars compacta neurons in Parkinson's disease.* Acta Neuropathol, 1994. **87**(4): p. 343-8.

13. Derex, L. and P. Trouillas, *Reversible parkinsonism, hypophosphoremia, and hypocalcemia under vitamin D therapy.* Mov Disord, 1997. **12**(4): p. 612-3.

14. Boucher, B.J., W.G. John, and K. Noonan, *Hypovitaminosis D is associated with insulin resistance and beta cell dysfunction.* Am J Clin Nutr, 2004. **80**(6): p. 1666; author reply 1666-7.

15. Evatt, M.L., et al., *Prevalence of vitamin d insufficiency in patients with Parkinson disease and Alzheimer disease.* Arch Neurol, 2008. **65**(10): p. 1348-52.
