# Supplementary material for: Elucidating the Relationship Between Diabetes Mellitus and Parkinson’s Disease Using 18F-FP-(+)-DTBZ, a Positron-Emission Tomography Probe for Vesicular Monoamine Transporter 2
Source: Front Neurosci. 2020 Jul 14;14:682. doi: 10.3389/fnins.2020.00682 (PMC7372188; doi:10.3389/fnins.2020.00682)
Supplement: Supplementary file 2 [file Table_2.docx]

**Table 1.** Recent studies on the correlation between diabetes mellitus (DM) and Parkinson’s disease (PD)

| **Study** | **Study design** | **Sample size** | | **Main results** |
| --- | --- | --- | --- | --- |
| Hu et al., 2007 [[1](#_ENREF_1)] | Cohort | | PD: 633 Controls: 51,552 | T2DM is associated with an increased risk of PD. |
| Moran et al., 2008 [[2](#_ENREF_2)] | Meta-analysis | | N/A | Shared biological pathways between PD, T2DM, cancer, and inflammation. |
| D’ Amelio et al., 2009 [[3](#_ENREF_3)] | Case-control | | PD: 318 Controls: 318 | Inverse association between PD and DM preceding PD onset. |
| Palacios et al., 2011 [[4](#_ENREF_4)] | Case-control | | PD: 1931 Controls: 9651 | T2DM is associated with an increased risk of PD, especially younger-onset PD. |
| Xu et al., 2011 [[5](#_ENREF_5)] | Cohort | | DM: 21,611 Controls: 267,051 | T2DM is associated with an increased risk of PD. |
| Bosco et al., 2012 [[6](#_ENREF_6)] | Case-control | | PD+dementia: 53 PD: 57 | Insulin resistance is associated with an increased risk of dementia in PD. |
| Sun et al., 2012 [[7](#_ENREF_7)] | Case-control | | DM: 603,416 Controls: 472,118 | DM is associated with an increased risk of PD onset. |
| Wahlqvist et al., 2012 [[8](#_ENREF_8)] | Case-control | | DM: 64,166 Controls: 698,587 | T2DM is associated with an increased risk of PD. Metformin-sulfonylurea therapy reduces the risk of PD. |
| Yue et al., 2016 [[9](#_ENREF_9)] | Meta-analysis | | Based studies: 7  Sample total: 1,761,632 | Diabetes is associated with an ~38% increase in the risk of PD. |
| De Pablo-Fernandez et al., 2017 [[10](#_ENREF_10)] | Cohort | | PD: 79  Controls: 4919 | Diabetes duration might be an important factor in the association of PD and diabetes. |
| De Pablo-Fernandez et al., 2018 [[11](#_ENREF_11)] | Cohort | | DM: 2,017,115 Controls: 6,173,208 | Significantly elevated rates of PD following T2DM. |
| Pagano et al., 2018 [[12](#_ENREF_12)] | Case-control | | PD+DM: 25 PD without DM: 25 DM: 14 Controls: 14 | DM may predispose toward a Parkinson-like pathology and, when present in patients with PD, can induce a more aggressive phenotype. |

T2DM, type 2 diabetes mellitus

1. Hu, G., et al., *Type 2 diabetes and the risk of Parkinson's disease.* Diabetes Care, 2007. **30**(4): p. 842-7.

2. Moran, L.B. and M.B. Graeber, *Towards a pathway definition of Parkinson's disease: a complex disorder with links to cancer, diabetes and inflammation.* Neurogenetics, 2008. **9**(1): p. 1-13.

3. D'Amelio, M., et al., *Diabetes preceding Parkinson's disease onset. A case-control study.* Parkinsonism Relat Disord, 2009. **15**(9): p. 660-4.

4. Palacios, N., et al., *Obesity, diabetes, and risk of Parkinson's disease.* Mov Disord, 2011. **26**(12): p. 2253-9.

5. Xu, Q., et al., *Diabetes and risk of Parkinson's disease.* Diabetes Care, 2011. **34**(4): p. 910-5.

6. Bosco, D., et al., *Dementia is associated with insulin resistance in patients with Parkinson's disease.* J Neurol Sci, 2012. **315**(1-2): p. 39-43.

7. Sun, Y., et al., *Risk of Parkinson disease onset in patients with diabetes: a 9-year population-based cohort study with age and sex stratifications.* Diabetes Care, 2012. **35**(5): p. 1047-9.

8. Wahlqvist, M.L., et al., *Metformin-inclusive sulfonylurea therapy reduces the risk of Parkinson's disease occurring with Type 2 diabetes in a Taiwanese population cohort.* Parkinsonism Relat Disord, 2012. **18**(6): p. 753-8.

9. Yue, X., et al., *Risk of Parkinson Disease in Diabetes Mellitus: An Updated Meta-Analysis of Population-Based Cohort Studies.* Medicine (Baltimore), 2016. **95**(18): p. e3549.

10. De Pablo-Fernandez, E., et al., *Association between Parkinson's disease and diabetes: Data from NEDICES study.* Acta Neurol Scand, 2017. **136**(6): p. 732-736.

11. De Pablo-Fernandez, E., et al., *Association between diabetes and subsequent Parkinson disease: A record-linkage cohort study.* Neurology, 2018. **91**(2): p. e139-e142.

12. Pagano, G., et al., *Diabetes mellitus and Parkinson disease.* Neurology, 2018. **90**(19): p. e1654-e1662.
